# Chromosomal imbalance letter: Phenotypic consequences of combined deletion 8pter and duplication 15qter

**DOI:** 10.1186/1755-8166-6-24

**Published:** 2013-07-01

**Authors:** Frenny Sheth, Joris Andrieux, Stuti Tewari, Harsh Sheth, Manisha Desai, Pritti Kumari, Nidhish Nanavaty, Jayesh Sheth

**Affiliations:** 1 FRIGE’s Institute of Human Genetics, FRIGE House, Jodhpur Gam Road, Satellite, Ahmedabad, 380 015, India; 2 Laboratory of Medical Genetics, Jeanne de Flandre Hospital CHRU de Lille, Lille Cedex, France; 3 Institute of Genetic Medicine, Newcastle University, International Centre for Life, Newcastle upon Tyne, NE1 4EP, UK

**Keywords:** Chromosomal imbalance, 8p23 deletion, 15q23 duplication, *IGF1R*, *GATA4*, *MCPH1*

## Abstract

Exact breakpoint determination by oligonucleotide array-CGH has improved the analysis of genotype-phenotype correlations in cases with chromosome aberrations allowing a more accurate definition of relevant genes, particularly their isolated or combined impact on the phenotype in an unbalanced state. Chromosomal imbalances have been identified as one of the major causes of mental retardation and/or malformation syndromes and they are observed in ~2-5% of the cases. Here we report a female child born to non-consanguineous parents and having multiple congenital anomalies such as atrial septal defect and multiple ventricular septal defects, convergent strabismus, micropthalmia, seizures and mental retardation, with her head circumference and stature normal for her age. Cytogenetic study suggested 46,XX,add(8)(p23). Further analysis by array-CGH using 44K oligonucleotide probe confirmed deletion on 8p23.3p23.1 of 7.1 Mb and duplication involving 15q23q26.3 of 30 Mb size leading to 46,XX,der(8)t(8;15)(p23.3;q23)pat.arr 8p23.3p23.1(191,530-7,303,237)x1,15q23q26.3(72,338,961-102,35,195)x3. The unique phenotypic presentation in our case may have resulted from either loss or gain of a series of contiguous genes which may have resulted in a direct phenotypic effect and/or caused a genetic regulatory disturbance. Double segmental aberrations may have conferred phenotypic variability, as in our case, making it difficult to predict the characteristics that evolved as a result of the global gene imbalance, caused by the concomitant deletion and duplication.

## Introduction

Chromosomal rearrangements are frequently observed in patients with multiple congenital anomalies (MCA), dysmorphism and developmental delay with/without mental retardation [1-3]. Inheritance of a balanced translocation from either or both parents is often responsible for structural chromosomal defects leading to segmental duplication or deletion of the chromosome pair in an affected individual [4]. The paradigm shift in diagnostics with the implementation of next generation *in silico* softwares and array based comparative genomic hybridization (aCGH) technology, chromosome breakpoint determination, analyses of critical regions involved in genetic disorders and copy number evaluation has helped to correlate chromosomal region alteration and the resulting phenotype [5,6].

Segmental deletion of chromosome 8p [7,8] and duplication involving chromosome 15q [9,10] independently are well characterized and accurately compared with the clinical features seen in the affected individuals. However, segmental aneusomies simultaneously covering large regions on both the chromosomes, leading to a phenotypic presentation, have rarely been described.

Partial deletion of 8p23 is a relatively frequent deletion syndrome characterized by major congenital anomalies, especially congenital heart defects, seizures, behavioral abnormalities and postnatal growth deficiency [8]. Facial dysmorphism may be subtle and mental retardation less severe than in those with deletions associated with more proximal breakpoints [8].

Duplication of the distal long arm of chromosome 15 has been reported in various studies, which mainly highlighted it as an overgrowth syndrome characterized by prenatal overgrowth, macrocephaly, tall stature and craniosynostosis [9]. However, such cases exclusively involve the distal most segment i.e. 15q25. It has been proposed by Roggenbuck et al. 2004, that the aforementioned distal 15q trisomy syndrome may be the result of the disruption of the gene linked to 15q25 region, rather than partial trisomy for the region. Their study reported 3 cases sharing features like ptosis, small size and developmental delay [10].

Here we present a female child with deletion 8p23.3p23.1 as well as duplication 15q23q26.3. The aberrant chromosome 8 was inherited from the phenotypically normal father who was the carrier of a balanced translocation 46,XY,t(8;15)(p23;q23). We describe her phenotype, at birth, at 4 years and 8 years of age. In addition, we describe the phenotypic consequences of the concomitant effect of segmental deletion and duplication of the same.

### Clinical presentation

A 4 year-old female child was referred for congenital heart defects, dysmorphic facial features and developmental delay evident since her birth. She was the first child born to a non-consanguineous couple. During pregnancy, her mother had developed oligohydramnios and pre-eclampsia in the third trimester. She had a history of two spontaneous 1^st^ trimester abortions, one before and one after the birth of proband. The baby was born full term by caesarian section. Her birth weight was 3.5 kg and she did not cry at birth (Apgar score 4–6). She received oxygen for respiratory distress in the NICU for 6 days. Initially, she had feeding difficulties but eventually started breastfeeding at 1½ month. Loud holosystolic murmur was evident from the 2^nd^ day of life, and the 2D echocardiograph showed atypical muscular atrial septal defect (ASD) and multiple ventricular septal defects (VSD), with no patent ductus arteriosus (PDA). TORCH investigations were normal.

**Figure 1:**
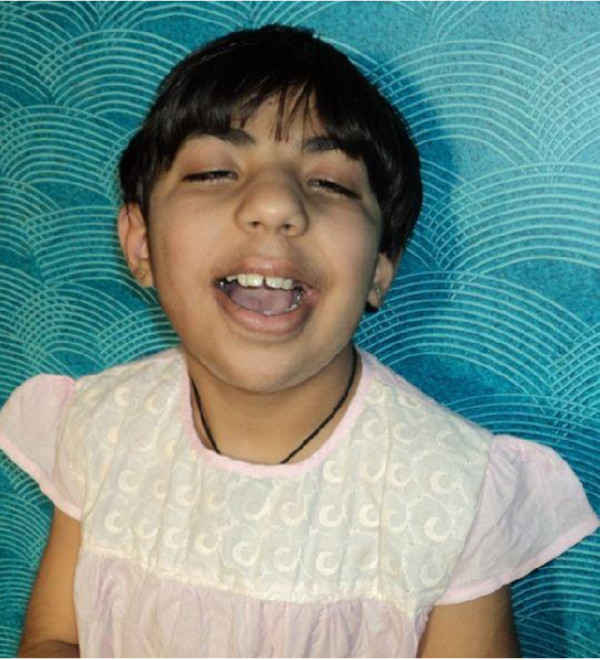
Clinical presentation of the proband at 8 years of age.

At the age of 4 years, the head circumference was 47 cm (10^th^ percentile), height 94.5 cm (15^th^ percentile) and weight 14.5 kg (25^th^ percentile) [11]. The prominent dysmorphic features were ptosis, downward slanted palpebral fissure, microphthalmia, left convergent strabismus, wide nasal base, long philtrum, open mouth, low set ears, short neck, micrognathia, puffy cheeks, short fingers, bilateral 1^st^ incurved finger and absence of thenar eminence [Figure 1]. Developmental delay was evident from the first year as social smile was absent even at 6 months. She learnt sitting at 10 months and walking at 20 months. Speech was absent and her developmental quotient at presentation was of a 2 year old child. She had 3 attacks of partial seizures during the first 20 months and was kept on carbamazepine 150 mg/day and sodium valproate 100 mg/day till she was 5 years old. Since then, she has been free of epileptic symptoms. Sequential complete hemogram was suggestive of chronic iron deficiency anemia with hemoglobin levels of 8–8.5 gm%. 2D echocardiograph again at 3 years showed multiple small muscular VSDs and a single ASD of 4.2 × 7.0 mm.

On re-examination at the age of 8 years, the head circumference was 49.5 cm (10^th^ percentile), height 120 cm (25^th^ percentile) and weight 28 kg (75^th^ percentile) [11]. Mental retardation was evident. She could not speak but responded to commands. The bladder and bowel control had not yet been achieved. Apart from these, no other developmental abnormalities were noted. She could carry out routine activities independently. It is to be noted that she had normal for age head circumference, height and weight.

## Results

### Metaphase chromosome analysis

**Figure 2:**
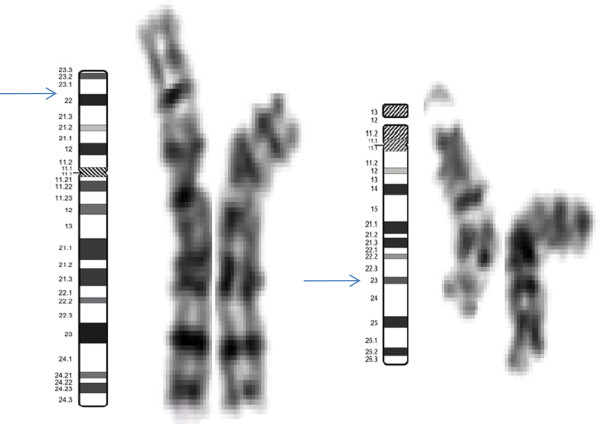
Partial karyotype of the father showing breakpoints on both derivative chromosomes.

After obtaining institutional ethical committee approval and informed written consent form, metaphase chromosome preparations were obtained from PHA stimulated lymphocyte cultures according to the standard procedure with slight modifications [12]. Chromosome analysis was carried out by GTG-banding at 550-band level according to ISCN 2013 nomenclature in both patient and parents (50 metaphases, each). Patient’s karyotype pattern showed additional genetic material on the short arm of #8p i.e.46,XX,add(8)(p23). Parental chromosomal investigation revealed that the father was a carrier of a balanced translocation 46,XY,t(8;15)(p23;q23) [Figure 2]. This suggests that the child had inherited der(8) from the father thus leading to an unbalanced genetic makeup.

### Array-CGH

**Figure 3:**
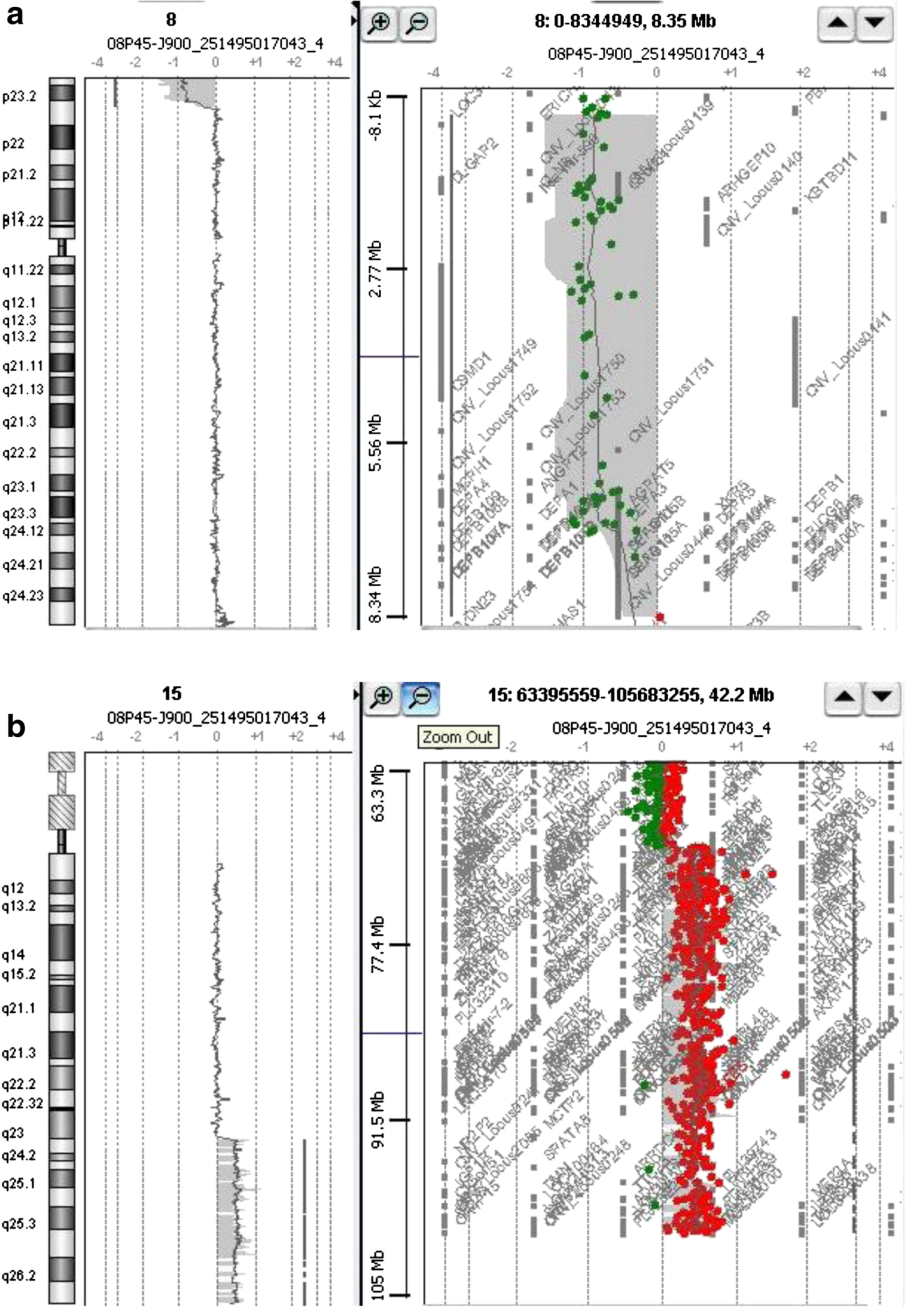
**Breakpoint characterization by 44K oligonucleotide array-CGH. a**: 7.1 Mb deletion at 8p [arr 8p23.3p23.1(191,530-7,303,237)x1] and **b**: 30 Mb duplication at 15q [arr 15q23q26.3(chr15:72,338,961-102,351,195)x3].

Genomic DNA was extracted from peripheral blood lymphocytes using standard SDS-proteinase K extraction method [13]. Extracted genomic DNA concentration was determined with NanoDrop ND-1000 spectrophotometer (NanoDrop Technologies, Berlin, Germany). Evaluation of gene copy number was performed by 44k oligonucleotide array-Comparative Genomic Hybridization (aCGH) by following manufacturer's recommendations (Human Genome CGH microarray 44B kit, Agilent Technologies Inc., Santa Clara, CA, USA). Female genomic DNA (Promega Corporation, Madison, WI, USA) was used as a sex-matched reference, which was analyzed with the CGH-analysis software v3.4 (Agilent Technologies Inc., Santa Clara, CA, USA) by applying Z-score segmentation algorithm with a window size of 10 points to identify chromosome aberrations. Analysis was performed using 3-points filter and 0.2 variation which lead to confirmation of partial deletion 8p region of 7.1 Mb [arr cgh 8p23.3p23.1(191,530-7,303,237)(hg19-NCBI build37)x1] and partial 15q duplication of 30 Mb [arr cgh 15q23q26.3 (chr15:72,338,961-102,351,195)x3], i.e. 46,XX,der(8)t(8;15)(p23.3;q23)pat.arr 8p23.3p23.1(191,530-7,303,237)x1,15q23q26.3(72,338,961-102,351,195)x3 [Figure 3a,b].

### Fluorescence *in situ* hybridization (FISH)

**Figure 4:**
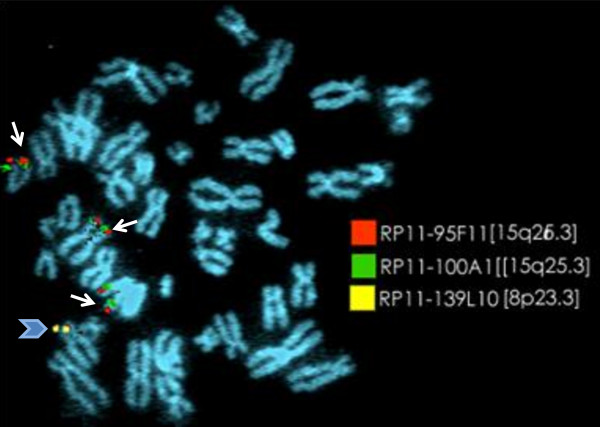
**Metaphase showing signals of both BAC clones RP11-95F11 and RP11-100A1 spanning 15q25.3 to 15q26.3 region on two normal #15 and on derivative #8p [thin arrow].** Whereas, clone RP11-139L10 covering 8p23.3 is seen only on normal #8p [broad arrow].

FISH analysis was performed using BAC clones RP11-139L10 covering 8p23 → pter, RP11-95F11 and RP11-100A1 spanning 15q23 → qter. Nick Translation Kit (Vysis, Abbott Molecular, USA) was used for labeling. The two clones - RP11-95F11 and RP11-100A1 were labeled with Spectrum Orange and Spectrum Green respectively whereas, third BAC clone was labeled using both fluorochromes in 1:1 ratio labeling. FISH signals were observed using Olympus BX-51 microscope (Olympus, Germany) and pseudo-coloring was carried out using Adobe Photoshop [Figure 4].

## Discussion

This case report presents a 4 year old girl who was diagnosed with double segmental chromosomal aberrations 46,XX,der(8)t(8;15)(p23;q23)pat. The derivative #8 was inherited from the father who was a carrier of balanced 8;15 chromosomal translocation which led to partial deletion of 8p23.3p23.1 and duplication of 15q23q26.3 in the child. Inheriting unbalanced chromosomal rearrangements that originated from a translocation event in either parent are often associated with mental retardation and/or congenital malformations [5].

The distal 8p deletion is of a relatively frequent occurrence with distinct clinical features [10]. Congenital heart defects, behavioral problems, mild to moderate mental retardation/developmental delay, strabismus and mild facial and digital anomalies are frequent phenotypic characteristics seen in these patients [9,14,15]. The severity of mental retardation, pre and postnatal growth deficiency including microcephaly and facial dysmorphism becomes more pronounced as the deletion site involves more proximal regions [15].

Cytogenetic evidence suggests that the haploinsufficiency of ≥1 gene located in 8p23 behaves as a dominant mutation, thus impairing heart differentiation and leading to a wide spectrum of congenital heart defects (CHDs) [8]. The gene responsible for the heart defects in this syndrome has been identified to be *GATA4* on 8p23.1 [14]. Haploinsufficiency of *GATA4* is thought to play a critical role in the development of these birth defects [16,17]. This data is in concordance with the findings in our patient who has atrial septal defect and multiple ventricular septal defects since birth.

Microcephaly is another feature frequently seen in patients with distal 8p deletion [15]. The *MCPH1* gene, mapped to chromosome 8p23, has been implicated to be a candidate gene for primary microcephaly [18,19]. Although, in our patient, microcephaly was not noted, this finding has also been reported by other studies [10,20]. This gene has been shown to have a high penetrance with large deletion, as has been detected in the present case. However, the possibility of non-penetrance and/or non-expressivity as the cause behind the absence of microcephaly cannot be entirely excluded [15]. The concomitant presence of duplication of distal 15q leading to a normal for age head circumference could also be a possibility.

Our proband had mild mental retardation with very little speech development and facial dysmorphic features like wide nasal base, puffy cheeks, low set ears, micrognathia are in concordance with other cases of 8p deletion syndrome [9]. But majority of her facial features, including those mentioned above, like ptosis, down slanted palpebral fissures, long philtrum, open mouth, mid crease in the lower lip are similar to the distinguishable facial characteristics seen in cases of distal duplication of 15q [21].

Since the first report of 15q22 → qter duplication by Fujimoto et al. 1974 [22], at least 71 other cases of similar or smaller distal 15q imbalances have been described [23]. The phenotype-genotype correlations observed in these cases resulted in the delineation of the 15q overgrowth syndrome, which is caused by the increased dosage of the genes that are present between 15q25-q26.3. The findings of overgrowth have reliably been associated with the extra copy of the *IGF1R* (insulin-like growth factor 1 receptor) gene located at 15q26.3 [23]. However, in our patient, no findings suggestive of overgrowth were observed. This can be explained by the fact that this girl has an associated duplication extending proximally till 15q23. Roggenbuck et al. 2004 and Zollino et al. 1999 described cases with duplication from 15q24-q26.3 and 15q25.1-qter respectively, which showed postnatal growth retardation and developmental delay [9,10]. Roggenbuck and co-workers in their study also proposed that the features of overgrowth observed in patients with distal duplication of 15q may not be specifically related to increased dosage of genes in this chromosomal region [10]. This could be the reason why the proband exhibits normal growth.

In summary, the phenotypic effects of 8p deletion were dominant over 15q duplication as depicted in Table 1. Both microcephaly and postnatal growth retardation reported in patients of 8p deletion syndrome and distal 15q duplication syndrome respectively were not observed in our patient. Combined chromosomal aberrations, as the present one, may confer phenotypic variability, thereby making it difficult to perform genotype-phenotype correlations [7]. As for all complex genetic features or disorders, phenotype is influenced by additional genetic and environmental factors. Also haploinsufficiency of the deleted gene(s) alone cannot explain this clinical variability, other modifying factors like single base pair mutations and/or polymorphisms of uninvolved loci may contribute [24].

**Table 1:** Comparison of phenotypes having partial 8p deletion and partial 15q duplication and its correlation with our case

**Clinical features**	**Partial 8p deletion**	**Our patient**	**Partial 15q duplication**
Mental retardation	+	++	+
Post natal growth deficiency	+	-	+
Microcephaly	+	-	+
Speech delay	+	+++	+
Congenital heart disease	+	+++	+\-
Seizures	+	+	+
Hypotonia	-	-	+
Behavioural abnormalities	+	-	-
Downslanted palpebral fissure	+/-	+	+
Strabismus	-	++	+
Ptosis	-	+++	+
Wide nasal base	+	++	+
Short neck	+	++	+
Bulbous nose	+	+	-
Low set ears	-	+	+
Puffy cheeks	+	+	+
Midline crease in lower lip	-	+	+
Thin upper lip	+	-	-
Long philtrum	-	+	+
High arched palate	-	+	+
Micrognathia	+	+	+
Pectus excavatum	-	-	+
Scoliosis	-	-	+
Depressed sternum	+	-	-
Short fingers	+	+	+
Inguinal and diaphragmatic hernia	+	-	-
Widely spaced nipples	+	++	-

To conclude, such correlations of defined phenotypic manifestations with the deletion or duplication of specific genes provides an opportunity to analyze gene-gene interactions and help to further unravel the intricacies of the human genome.

## Consent

Written informed consent was obtained from the parents for publication of this case report and accompanying images of the child. A copy of the written consent is available for review by the Editor-in-Chief of this journal.

## Competing interests

The authors declare that they have no competing interests.

## Authors’ contributions

MD and HS performed the cytogenetic studies. Molecular cytogenetic analysis was carried out by MD whereas clone selection and interpretation was carried out by FJ in the present case. ST, PK and NN collected the data relative to this case report. JA was involved in the array-CGH analysis. ST and PK drafted the paper. FJ and JS revised the manuscript for important intellectual content. All authors contributed to the finalizing of the manuscript. All authors read and approved the final manuscript.
